# Bioinformatics Approaches to the Understanding of Molecular Mechanisms in Antimicrobial Resistance

**DOI:** 10.3390/ijms21041363

**Published:** 2020-02-18

**Authors:** Pieter-Jan Van Camp, David B. Haslam, Aleksey Porollo

**Affiliations:** 1 Department of Biomedical Informatics, University of Cincinnati, Cincinnati, OH 45267, USA; vancampn@mail.uc.edu; 2 Division of Biomedical Informatics, Cincinnati Children’s Hospital Medical Center, Cincinnati, OH 45229, USA; 3 Division of Infectious Diseases, Cincinnati Children’s Hospital Medical Center, Cincinnati, OH 45229, USA; David.Haslam@cchmc.org; 4 Department of Pediatrics, University of Cincinnati, Cincinnati, OH 45267, USA; 5 Center for Autoimmune Genomics and Etiology, Cincinnati Children’s Hospital Medical Center, Cincinnati, OH 45229, USA

**Keywords:** antimicrobial resistance, antibiotic resistance genes, molecular mechanisms, bioinformatic analysis, prediction of antibiotic resistance

## Abstract

Antimicrobial resistance (AMR) is a major health concern worldwide. A better understanding of the underlying molecular mechanisms is needed. Advances in whole genome sequencing and other high-throughput unbiased instrumental technologies to study the molecular pathogenicity of infectious diseases enable the accumulation of large amounts of data that are amenable to bioinformatic analysis and the discovery of new signatures of AMR. In this work, we review representative methods published in the past five years to define major approaches developed to-date in the understanding of AMR mechanisms. Advantages and limitations for applications of these methods in clinical laboratory testing and basic research are discussed.

## 1. Introduction

Antimicrobial resistance (AMR) contributes to antibiotic (AB) treatment failure and increasing rates of mortality in various infectious diseases. Misuse of specific AB and overuse of broad spectrum AB are attributed to the emergence of AMR, which in some strains results in multidrug resistance (MDR). As MDR organisms become more common in clinical settings, fast and accurate assessment of antibiotic susceptibility is needed to start effective treatment as soon as possible [1]. However, not all molecular mechanisms underlying AMR are uncovered or understood yet, especially in Gram-negative bacteria. This means that currently the most reliable way for evaluating resistance to AB in clinical microbiology laboratories is to grow organisms in culture, expose them to various antibiotic concentrations, and assess the impact on growth. This approach can range from a day to several weeks, depending on the growth rate of the pathogen. Some species do not even grow in known culture media. This relatively time consuming process is not in line with current clinical demands on fast decision-making, and thus alternative methods for AMR evaluation based on underlying genetic and molecular information have been the shift in focus.

**Figure 1:**
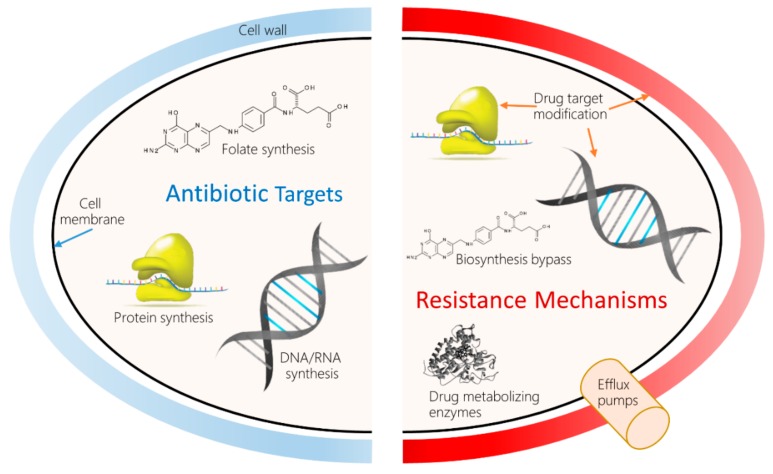
Antimicrobial drug targets and molecular mechanisms of antimicrobial resistance (AMR). **Left:** The most common classes of AB currently in use impede bacterial growth by inhibiting the biosynthesis of peptidoglycan, a main constituent of cell wall; disrupting the bacterial cell membrane; and inhibiting DNA replication, gene transcription and translation, and folate biosynthesis. **Right:** In turn, bacteria have developed many resistance mechanisms to these attacks, such as pumping the AB out of the cell, inactivating the drug using specialized enzymes, modifying the target structures to prevent interference, and bypassing the affected metabolic pathway.

Decades of research into various molecular mechanisms of AMR have revealed scores of antibiotic resistance genes (ARG), or bacterial gene variants associated with phenotypical resistance to AB. An example is the NCBI Bacterial Antimicrobial Resistance Reference Gene Database (NCBI Accession ID: PRJNA313047). Resistance can develop in many ways, depending on the target. Figure 1 shows a high-level overview of AB targets and the types of established bacterial AMR mechanisms to counteract the drugs.

The mechanism of action of many AMR genes or cellular pathways are already known. In some cases where the genotype–phenotype relationship is straightforward, gene-based prediction has found its way into clinical practice in the form of rapid molecular tests. An example is the detection of the mecA gene in *Staphylococcus aureus* to determine whether it is methicillin-resistant *S. aureus* (MRSA) and therefore requires modification of a typical antibiotic treatment approach. Unfortunately, many types of resistance are more complex as there is often incomplete penetrance of the ARG, and thus simple association with phenotypic resistance, such as mecA, is not possible, especially when trying to generalize ARG across multiple species or while evaluating multiple AB at once [2].

With the advent of whole genome sequencing (WGS) permitting quick access to de novo sequenced genomes of pathogens and the collection of sufficiently large datasets of clinical isolates, it is now plausible to employ advanced bioinformatics approaches (e.g., machine learning) to gain new insights in the more complex molecular mechanisms of AMR. These approaches can evaluate many samples at once by exploring different types of data (e.g., genomics- or metabolomics-based) and reveal new insights previously not attainable.

In the past few years, the field of biomedical AMR prediction has evolved rapidly. In this review, we summarize the current state of bioinformatics methods for the discovery of AMR molecular mechanisms and discuss advantages and limitations of machine leaning (ML) models in the prediction of AMR and their use in clinical settings.

## 2. Bioinformatics Approaches to the Analysis and Prediction of Antimicrobial Resistance

Table 1 lists the methods that are reviewed here, chosen either to illustrate the evolution of methodology or as representatives of orthogonal approaches to predicting AMR. Methods are listed in the chronological order of their original publication, but will subsequently be grouped and discussed per approach they employ.

### 2.1. Approach 1: Identification of Known Genomic Signatures of AMR from WGS Data

The first approach to predicting the AMR is to analyze WGS (e.g., Illumina sequencing) data by identifying the presence of known ARG or gene variants. Some genes are endogenous to a given species/strain, while others reside on mobile genetic elements (plasmids) that can be shared between species. The corresponding binary (presence or absence) or real number (abundance) vectors that represent such fixed sets of genes and their mutations can be subsequently used for training ML models and predicting newly sequenced samples.

TypeWriter uses BLASTn to match assembled genomic contigs to 24 ARG and 120 mutations associated with resistance in *S. aureus* [3]. The assembly step followed by comparison with a reference strain of the bacterium ensures the quality control of sequencing and genome assembly. This makes the overall process slow, raising questions about applicability of the workflow to a mixture of isolated strains and to other species. PointFinder uses BLASTn to map raw reads to a set of 16 ARG and their mutations [8]. The main contribution to the field of this method is to expand and validate this approach in relation to three species: *E. coli*, *C. jejuni*, and *S. enterica*. PhyResSE [5] applies BWA-MEM [16], a faster than BLASTn sequence aligner, to map shotgun read sequences to a reference genome of *M. tuberculosis* (strain H37Rv) in order to identify what lineage a given isolate represents from a pre-compiled catalog of 92 lineages with known resistance phenotype. The method eliminates the need for genome assembly step but requires a number of additional steps for assuring the quality of called variants, including FastQC [17], Qualimap [18], and Genome Analysis Toolkit (GATK) [19,20]. PhyResSE still suffers from the same applicability issues mentioned for TypeWriter above. Mykrobe employs an alignment-free approach by using de Bruijn graphs to build a library of reference strains of *S. aureus* and *M. tuberculosis* susceptible and resistant to multiple AB [6]. Then, raw reads of a newly sequenced sample are used to build new de Bruijn graphs that are subsequently compared to the reference to make a call on drug susceptibility. This method is faster than the previous three and can deal with a mixture of strains in one sequenced sample. The authors of the method also assessed its applicability to other staphylococcal species. The culmination of this bioinformatics approach is represented by the CRyPTIC consortium report that performed analysis at impressive scale of 10209 samples from 23 collections of *M. tuberculosis* complex isolates, then using only 9 ARG along with mutations to predict susceptibility to 4 AB [12].

Collectively, these methods operate with a predefined panel of ARG and associated mutations. They can yield accurate results with high confidence applicable in clinical settings. The sequencing of a bacterial genome has become fast (about 24 h and decreasing) and does not require several days of additional growth in vitro, as is required for traditional laboratory AMR testing. This could decrease the time to start effective AB therapy, thereby improving patient outcomes. Of note, most of these methods have been developed for only one specific pathogen (often *M. tuberculosis*, as this is very hard to grow), and it is not clear yet if this type of model would work equally well on other species with potentially more complex AMR mechanisms.

Furthermore, as these models are built for predictive power, they often have less potential for new biological insights into the underlying AMR mechanisms as they solely rely on already known genes and variants. Moreover, their applicability to other less studied species (especially Gram-negative bacteria with complex ARG patterns or many unknown variants) and to metagenomics samples (e.g., blood or stool samples) could be low.

### 2.2. Approach 2: Identification of AMR Signatures from Gene Expression Data

The second approach to the AMR analysis and prediction is to study changes in gene expression of the isolate upon drug treatment. Suzuki and colleagues induced *E. coli* evolution in the lab under pressure of 11 AB with different concentrations to obtain strains resistant to these drugs with various degrees, measured in terms of minimum inhibitory concentrations (MIC) [4]. Subsequent gene expression analysis (based on microarray) allowed them to study dynamic compensatory mechanisms in the pathogen. They found 8 genes (*acrB*, *ompF*, *cyoC*, *pps*, *tsx*, *oppA*, *folA*, and *pntB*) whose expression levels allowed for a linear regression model achieving correlation with experimentally determined MIC with R^2^ ranging from 0.54 to 0.75 per drug. Darnell and colleagues studied transcriptional response of *E. faecalis* (strain JH2-2) to teixobactin [15]. Genome-wide transcriptome analysis was conducted using RNA-seq upon the AB treatment. 573 differentially expressed genes were identified, with enrichment for such upregulated pathways as peptidoglycan, teichoic acid, and cell wall exopolysaccharide biosynthesis. Subsequent comparison of the expression profile with transcriptional responses of *E. faecalis* to other cell wall targeting antimicrobials revealed the shared upregulated CroRS regulon of 219 genes. CroRS is the cell wall stress response two-component system (TCS). The corresponding deletion mutant (*ΔcroRS*) significantly increased susceptibility of the organism to teixobactin.

Overall, this approach represents great promise in studying AMR molecular mechanisms. We envision that besides known dynamic mechanisms, such as overexpression of genes targeted by AB and xenobiotic efflux transporters, new studies may reveal regulatory circuits responding to various toxic stimuli (i.e., AB) and other compensatory mechanisms. However, given the complexity of the experiments, and the long time required to generate and analyze the data, this approach is unlikely to be employed in clinical settings soon, especially given the current modest accuracy of prediction. Moreover, not all clinically important pathogens (and their strains) are as well annotated as *E. coli*, and identification of informative differentially expressed transcripts may further be delayed by the need of functional annotation to understand their role in AMR. The analysis of complex isolates like microbiome samples presents even bigger challenges, as these metatranscriptomics data need deconvolution before they can be useful in any research or prediction.

### 2.3. Approach 3: ARG Agnostic Identification of AMR Mechanisms via Pan-Genome Analysis

The third approach to elucidating the AMR mechanisms is gene agnostic and based on global genomic comparison of multiple strains with various susceptibility to different drugs. PanPhlAn compared 110 reference and 12 metagenomically detected strains of *E. coli*, both commensal and outbreak-related, with respect to their genomic content and gene expression profiles [7]. The comparison enabled the identification of genes associated with *E. coli* outbreaks that were subsequently characterized with Gene Ontology (GO) and mapped to genomic functional modules defined in the KEGG database [21]. Mahe and Tournound applied this approach to multiple strains of *M. tuberculosis* and *S. aureus*, but they went further than the former study and extracted any 31-mers of genomic sequences (including from noncoding regions) that were uniquely associated with given pairs of drugs and AB susceptibility levels [10]. After LASSO regression with L1 regularization, they were able to identify a handful of *k*-mers (*n* = 1 to 8 per AB; 22 total) to be used in linear regression models for each tested AB with performance on par with the state of the art methods, such as Mykrobe. After assembly of these *k*-mers into unitigs and aligning them to the annotated genome, all *k*-mers appeared to belong to 10 genes or RNA: the *embB* gene for ethambutol, *fabG1*—ethionamide, *katG* and *fabG1*—isoniazid, *rss* and *eis*—kanamycin, *gyrA*—ofloxacin, *rpoB*—rifampicin, and *rss* and *rpsL*—streptomycin. Kavvas and colleagues [11] generated a pan-genome of *M. tuberculosis* based on 1595 sequenced strains and employed additional information from the PATRIC database [22], including the lineage defining SNPs, AMR phenotypic, and geographic and other data. Using both pairwise association tests (Mutual Information, χ^2^, and ANOVA F-test) and a machine learning algorithm (Support Vector Machine, SVM), they were able to identify 24 new genetic signatures of AMR and reveal 97 epistatic interactions associated with 10 classes of resistance in *M. tuberculosis*. Subsequent mapping to available 3D structures of select proteins associated with AMR allowed the authors to pin point the clusters of mutations in these proteins in response to AB. Such additional insight may help one to better understand the AMR causality of alleles in future studies. The latest and most comprehensive work within this approach was published by Drouin and colleagues and covers 12 bacterial species and 56 drugs [13]. In addition to generating the collections of *k*-mers associated with susceptible or resistant strains, they applied rule-based ML algorithms—Classification and Regression Trees (CART) and Set Covering Machines (SCM). These rule-based models provide clues to the importance of specific *k*-mers in decision making, which may facilitate the discovery of new mechanisms of AMR in different organisms.

The pan-genome approach, while requiring a prior large investment in genomic sequencing and genome wide comparison, also holds great promise for clinical application. The major advantage of this approach is exploring whole genomes instead of just known AMR-related regions, potentially identifying new mechanisms or regulatory elements (e.g., via mutations in noncoding, possibly regulatory regions). The challenge of these methods for machine learning is that the input space is very large (e.g., whole genome) and detection of novel regions needs rigorous evaluation (e.g., lab experiments) in order to validate its importance. This is because association between certain genomic regions in samples with similar AMR can be purely statistical rather than causal, a concept known as overfitting of ML models. Furthermore, these methods often require large collections of strains to obtain informative insights in AMR, something that would be a challenge for under-studied pathogens.

### 2.4. Approach 4: Identification of AMR Mechanisms from Metabolomics Data

The last approach, orthogonal to those described above, takes advantage of metabolic profiling. Yang and colleagues treated *E. coli* with 3 AB and analyzed the changes in the metabolite profiles [14]. By combining the known metabolic pathways of the organism with experimentally measured boundaries of metabolite concentrations upon drug treatment, they were able to conduct flux balance analysis (FBA). Then, metabolite fluxes and metabolic pathways were fed to machine learning models to predict lethality of a given AB. These models also revealed mechanisms of action for different AB as to what metabolic pathways are affected and how the bactericidal action can be enhanced, for example, via limitation of adenine. Zampieri and colleagues used similar approach to grow *E. coli* lineages under pressure of various combinations of 3 AB, but they also altered carbon source in the culture media to force either respiratory or fermentative metabolism of glucose [9]. In addition to tracking the changes in metabolite concentrations between lineages, the authors sequenced whole genomes for mutations developed through AB-induced evolution and measured gene expression and protein abundance of the multidrug efflux pump gene *acrB*. It was observed that *E. coli* employs multiple mechanisms to compensate for the pressure from different antibiotics, either via mutations or expression of efflux pumps; it also depends on the carbon source and therefore metabolic state the organism is in. This observation further supports the notion that Gram-negative bacteria are more complex to study and predict in terms of genotype–phenotype in the context of AMR.

Collectively, this approach gives a new perspective on the understanding of AB action and response of bacteria to drugs. While it is far from being easily translated to clinical practice, this approach lays the foundation to improve drug treatment regimens and optimize combined therapies. Extension of this approach to other pathogenic organisms may present challenges, since not all species are as well studied in terms of metabolic pathways as the model organism *E. coli*.

## 3. Conclusions

Bioinformatics approaches to the AMR research can be broadly categorized as those that focus on fast and reliable predictions of AMR to be applied in clinical settings, and those that explore the underlying molecular mechanisms of AMR. The first category primarily stems from prior knowledge of AMR molecular mechanisms, and may better generalize patterns of genotype–phenotype relationships. With the advent of WGS technologies, approaches in the first category tend to utilize sequencing data in order to identify the presence of known signatures of AMR, such as resistance genes or specific alleles known to elicit resistance. Approaches from the second category employ more difficult-to-generate data or complex datasets, such as pan-genomes, gene expression profiles, metabolomics, or biological pathways. The latter approaches are more geared towards revealing novel resistance genes, epistatic interactions related to AMR, underlying regulatory mechanisms, or new drug targets.

Each considered approach possesses certain limitations, discussed in the corresponding sections, and they should be considered when building and evaluating new prediction models. Nevertheless, the combination of modern molecular methods and powerful ML algorithms holds great promise for the understanding of AMR at the molecular level and for improved clinically relevant predictions leading to a more personalized AB use and more favorable clinical outcomes.

## Figures and Tables

**Table 1:** Representative bioinformatics methods for AMR analysis and prediction *.

Method	Targeted Species	Number of AB	Type of Prediction; Category **	Input Data	Feature Space
TypeWriter by Gordon et al., 2014 [3]	*Staphylococcus aureus*	12	Binary SvR classification; CA	WGS	24 ARG and their mutations
By Suzuki et al., 2014 [4]	*Escherichia coli*	11	Regression, MIC; EA	Gene expression (Microarray)	8 differentially expressed genes
PhyResSE by Feuerriegel et al., 2015 [5]	*Mycobacterium tuberculosis*	NS	Binary SvR classification; CA	WGS	11 ARG and a catalog of SNPs defining 92 strains
Mykrobe by Bradley et al., 2015 [6]	*S. aureus*, *M. tuberculosis*	12	Binary SvR classification; CA	WGS	Curated list of known ARG and their mutations (*n* = NS)
PanPhlAn by Scholz et al., 2016 [7]	*E. coli*	NA	Binary SvR classification; PCU	Metagenomics, Meta-transcriptomics	122 strains
PointFinder by Zankari et al., 2017 [8]	*E. coli,* *Campylobacter jejuni,* *Salmonella enterica*	11	Binary SvR classification; CA	WGS	16 ARG and their mutations
By Zampieri et al., 2017 [9]	*E. coli*	3	NA; EA	WGS, Metabolomics	Metabolites, Mutations, 12 lineages
By Mahe and Tournoud, 2018 [10]	*S. aureus,* *M. tuberculosis*	7	Logistic regression; PCU	WGS	1 to 8 genomic k-mers per AB (k = 31)
By Kavvas et al., 2018 [11]	*M. tuberculosis*	13	NA; PCU	WGS, protein structures	1595 strains
CRyPTIC consortium, 2018 [12]	*M. tuberculosis*	4	Binary SvR classification; CA	WGS	9 ARG and their mutations
By Drouin et al., 2019 [13]	*A. baumannii* *E. faecium* *E. coli* *K. pneumoniae* *M. tuberculosis* *N. gonorrhoeae* *P. difficile* *P. aeruginosa* *S. enterica* *S. aureus* *S. haemolyticus* *S. pneumoniae*	56	Multi-class classification; PCU	WGS	Genomic k-mers
By Yang et al., 2019 [14]	*E. coli*	3	NN regression; EA	Metabolomics	Metabolites, Metabolic networks, FBA
By Darnell et al., 2019 [15]	*Enterococcus faecalis*	1	NA; EA	Gene expression (RNA-seq)	573 differentially expressed genes

* NA—not applicable; NS—not specified; NN—neural networks; SvR—susceptible versus resistant. ** Categories: CA—clinically applicable; PCU—primed for clinical use; EA—exploratory approaches.

## Abbreviations

AB	antibiotic
AMR	antimicrobial resistance
ARG	antibiotic resistance gene
FBA	flux balance analysis
MIC	minimum inhibitory concentration
ML	machine learning
SNP	single nucleotide polymorphism
WGS	whole-genome sequencing
